# FASER: a tool for vectorial point spread function simulation with applications in stimulated emission depletion microscopy

**DOI:** 10.1117/1.NPh.12.1.017801

**Published:** 2025-02-12

**Authors:** Johannes Roos, Stéphane Bancelin, U. Valentin Nägerl

**Affiliations:** a Johannes Kepler University, Institute of Anatomy and Cell Biology, Linz, Austria; b University of Bordeaux, Interdisciplinary Institute for Neurosciences, CNRS, UMR 5297, Bordeaux, France; c IOGS - CNRS - University of Bordeaux, Numerical and Nanosciences Photonics Laboratory (LP2N), UMR 5298, Talence, France; d University Medical Center Göttingen, Department of Anatomy and Cell Biology, Göttingen, Germany

**Keywords:** optics, point spread function calculations, vectorial simulation, optical aberrations, microscopy, Python software, stimulated emission depletion, Napari

## Abstract

We introduce FASER, a software package designed to simulate the excitation point spread functions (PSFs) of microscopes. It is written in Python as a plugin for the open-source platform Napari. Using a full-vectorial computational approach to simulate the electromagnetic fields within the focal region makes precise predictions and allows detailed analyses of excitation PSFs. FASER is intended as a pedagogical tool enabling users to explore the impacts of various geometrical and optical parameters of practical importance on the performance of the microscope. It supports the modeling of complex beam profiles, including donut and bottle-shaped beams, which are commonly used in advanced microscopy techniques such as stimulated emission depletion (STED) microscopy. Through specific simulations and accessible illustrations, we showcase FASER’s capabilities in capturing characteristic features of STED microscopy, making it a practical resource for researchers and students in optical microscopy to explore and optimize high-resolution imaging techniques.

## 1 Introduction

Optical microscopy occupies a central place in the field of biophotonics, with new imaging technologies enabling significant biological breakthroughs. As technology keeps progressing, conceptual and technical improvements push the limits of optical microscopy in terms of spatial and temporal resolution,^1^ sensitivity, specificity, minimal invasiveness, and depth penetration,^2^ heralding the era of quantitative multiscale optical biology.^3^^,^^4^

Optical microscopy typically relies on the use of tightly focused light fields. In most cases, image quality (e.g., SNR, spatial resolution, sensitivity) depends strongly on the size and shape of the point spread function (PSF) of the microscope. Hence, precise control of the optical properties of focused beams (intensity, polarization, and phase) is critical for designing optical microscopes with high imaging performance. Beyond the optical beam properties, the PSF can be strongly affected by heterogeneities in the optical path of the microscope and the sample.^5^^–^^7^

How to focus light in the best way has drawn considerable interest for many decades. Over the years, extensive literature on analytical and numerical models to compute the diffracted light distribution has emerged. Because high-resolution optical microscopy commonly employs high numerical aperture (NA) objectives, a fully vectorial model is required to describe the focused light field. The corresponding equations are well established,^8^^,^^9^ notably through the work of Török,^10^^,^^11^ with analytical or numerical solutions investigated in specific configurations.^12^^–^^14^

However, a general analytical solution has not yet been derived. Due to the complexity of numerical calculations, various approximations are often used, limiting the simulation of the PSF to specific cases. In particular, paraxial approximation (small angles with respect to the optical axis), scalar approximation (neglected polarization of light), and aberration-free conditions are commonly assumed.

Here, we introduce FASER, an open-source Python software package designed to simulate the PSF of an optical microscope using fully vectorial calculations. It allows users to set the intensity profile, polarization, and phase mask of the incident laser beam, as well as to modify the geometry of the focusing medium. This tool was designed for pedagogical purposes, letting users simulate the effect of key optical and geometrical parameters on the PSF of the microscope. Specifically, FASER was expressly designed with *in vivo* experiments in mind, to make it possible to simulate the impact of a cranial window (e.g., refractive index, thickness, size, and tilt) and thus to help optimize the key parameters for an optical imaging protocol.

Developed as a plugin for Napari,^15^ FASER provides a graphical user interface (GUI) that allows running simulations directly without any coding skills. In addition, a batch mode functionality exists for most parameters, letting users vary parameters systematically to determine their impact and tolerance range.

In this work, we illustrate the potential of FASER to simulate shaped PSFs, such as those used in STED and reversible saturable optical fluorescence transition (RESOLFT) microscopy, in the context of *in vivo* microscopy, including a cranial window and a coverslip. We investigate the effects of common cases of misalignments and optical aberrations on the STED PSF and the resulting effective fluorescence PSF.

## 2 Theoretical Background

### 2.1 Light Distribution in the Focal Region

**Fig. 1:**
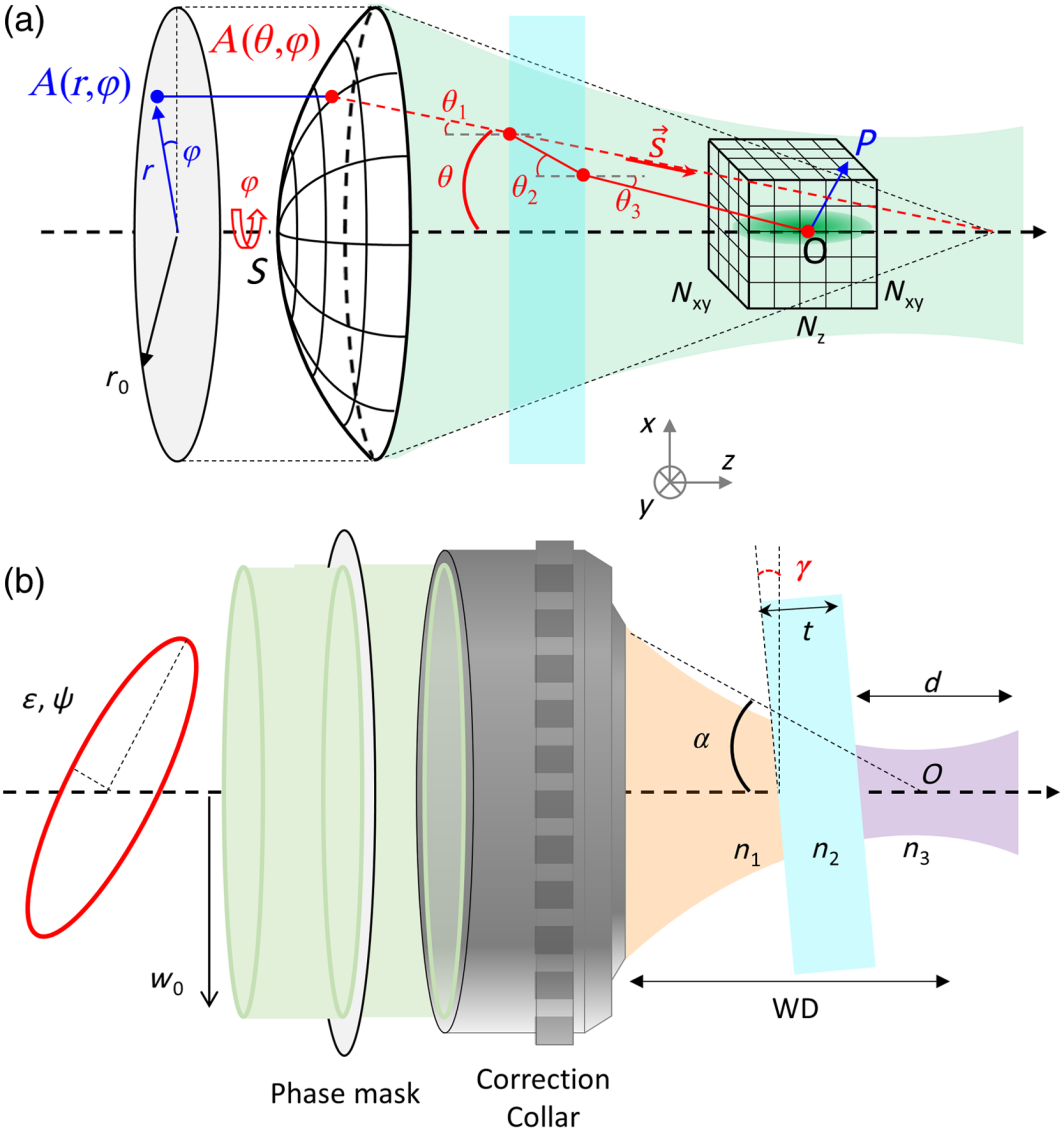
Schematic of the geometry used for the simulations. (a) Using vectorial diffraction theory, the incident pupil field A(r,φ) is transformed into a spherical wavefront through the objective lens and then propagated to the focal spot. The focal intensity is calculated at any point P in the vicinity of the focus O. The focalization process is computed through the stratified media consisting of immersion liquid, coverslip, and sample. (b) The ellipticity, intensity profile, and potential aberrations in the input beams are determined before the objective. A phase mask can also be added in the context of beam shaping, as in the case of STED microscopy.

In an optical imaging system, if the NA of the focusing lens is relatively high (NA ≥0.7), the approximations used in scalar diffraction theory, such as the paraxial approximation, Kirchhoff boundary condition, and Fresnel or Fraunhofer approximation are no longer valid.^8^ In the 1950s, Richard and Wolf proposed a comprehensive mathematical representation of the electromagnetic field (EM) distribution in the focal region of a high NA objective lens.^16^^,^^17^ A precise investigation of the validity range of this integral representation has been proposed in the literature.^13^^,^^18^ The main assumptions of this theory can be summarized as follows:

• The beam at the exit pupil has a spherical wavefront with a radius f, the focal length of the objective.
• Each diffracted light ray is considered as a plane wave propagating toward the geometrical focal point of the lens, indicated by the wave vector k.
• The observation point is far from the exit pupil plane [SP≫λ, see Fig. 1(a)], and the pupil size is large compared with the wavelength (f sin α≫λ).

Following this formalism, known as vectorial diffraction theory, the electric field (E) in an arbitrary point P(x,y,z) in the focal region of a high NA objective lens results from the superposition of all diffracted plane waves emerging from the exit pupil of the lens within the solid angle Ω^8^^,^^10^^,^^17^

$$E(x,y,z)=-\frac{ikf}{2\pi }\iint_{\Omega }\frac{a(s_{x},s_{y})}{s_{z}}e^{iks.r}ds_{x}\;ds_{y},$$ (1)

where k is the wavenumber, $s=(s_{x},s_{y},s_{z})$ is a dimensionless unit vector along each ray from the objective pupil toward the geometrical focal point O, a the complex amplitude of the incident laser beam after the objective, and r is the position vector of point P(x,y,z). For the sake of clarity, it is more convenient to express the wave vector s and pupil function in spherical coordinates. In this case,

$$\left\{ \begin{array}{l} s=(s_{x},s_{y},s_{z})=(\sin\;\theta \;\cos\;\varphi ,\;\sin\;\theta \;\sin\;\varphi ,\;\cos\;\theta )\\ d\Omega =\frac{ds_{x}ds_{y}}{s_{z}}=\sin\;\theta d\theta \;d\varphi \end{array}.\right.$$ (2)

Considering the geometry depicted in Fig. 1, the diffraction integral can be expressed as

$$E^{\left(1\right)}(x,y,z)=-\frac{ik_{0}f}{2\pi }\int_{0}^{\alpha }\int_{0}^{2\pi }\Lambda^{\left(1\right)}(\theta ,\varphi )A^{\left(0\right)}(\theta ,\varphi )e^{ik_{0}n(x\;\sin\;\theta \;\cos\;\varphi +y\;\sin\;\theta \;\sin\;\varphi +z\;\cos\;\theta )}\;\sin\;\theta d\theta \;d\varphi ,$$ (3)

with $k_{0}$ as the wavenumber in the vacuum, n as the refractive index in the medium, α=arcsin(NA/n) as the marginal ray angle of the objective (semi-aperture angle), and $\Lambda^{\left(1\right)}(\theta ,\varphi )$ as an operator describing the transformation of the complex vector field $A^{\left(0\right)}(\theta ,\varphi )$ incident at the back aperture of the objective lens into the field $A^{\left(1\right)}(\theta ,\varphi )$ on Ω after refraction by the objective lens. The action of this operator is explained in detail in Secs. 3.2.1 and 3.2.2.

### 2.2 Focusing Through a Glass Coverslip

In optical microscopy, light is typically focused on the sample through a layer of water or oil as an immersion medium and a thin coverslip for protection of the sample. To investigate this configuration, Richard and Wolf’s solution was extended by Törok et al.^10^ to account for a planar dielectric interface and further generalized to stratified media.^11^ Although this framework can describe an arbitrary number of layers with different refractive indices, only two interfaces are necessary to describe a glass coverslip placed between the sample and immersion media (Fig. 1).

In this case, the focal field in the sample, after the coverslip can be written

$$E^{\left(3\right)}(x,y,z)=-\frac{ikf}{2\pi }\int_{0}^{\alpha }\int_{0}^{2\pi }\Lambda^{\left(3\right)}(\theta ,\varphi )A^{\left(0\right)}(\theta ,\varphi )e^{ik_{0}n_{1}(x\;\sin\;\theta_{1}\;\cos\;\varphi +y\;\sin\;\theta_{1}\;\sin\;\varphi )}\;e^{ik_{0}n_{3}z\;\cos\;\theta_{3}}e^{ik_{0}(n_{3}d\;\cos\;\theta_{3}-n_{1}(t+d)\cos\;\theta_{1})}\;\sin\;\theta d\theta \;d\varphi ,$$ (4)

with d as the depth in the sample, t as the thickness of the coverslip, and $n_{l}$ and $\theta_{l}$ as the refractive indices and incident angles in the immersion medium (1), coverslip (2), and sample (3), respectively. These parameters are determined according to Snell’s law.

Note that $\Lambda^{\left(3\right)}(\theta ,\varphi )$ describes the polarization conversion in a heterogeneous medium after the objective aperture, including Fresnel coefficients of reflection and transmission through the coverslip. Notably, it includes the different apodization for s and p polarization.

The third exponential term corresponds to aberrations induced along the optical path by the coverslip and the biological sample. Notably, it is independent of the polar angle $\theta_{1}$ and thus describes spherical aberrations. The effect of a correction collar can hence be simulated, assuming perfect correction mechanisms, by applying the inverted aberration terms

$$\Phi_{\mathrm{corr}}^{\left(3\right)}=n_{3}d\;\cos\;\theta_{3}-n_{1}(t+d)\cos\;\theta_{1}.$$ (5)

Notably, the case of a coverslip-corrected microscope objective can be computed by simply setting the correction collar to a defined value.

## 3 FASER: Vectorial PSF Simulations

As highlighted in Eq. (4), the PSF in the focal region of a high NA objective lens depends on several parameters. Although these equations are well-known, performing full-vectorial simulations of the PSF in the focus of a high NA objective lens remains a complex task, and up to now, most freely available simulation tools only allow calculations for specific sets of configurations.

FASER is intended as a pedagogical tool for microscope developers and users, as well as optical aficionados, making it possible to explore the impact of numerous parameters related to the excitation beam and focusing geometry. This flexibility facilitates simulating PSFs that one encounters under real-world experimental conditions. The following subsections outline these parameters and how they are implemented in FASER.

### 3.1 Numerical Calculation

In principle, there is an infinite number of diffracted rays within the solid angle Ω that emerge from the lens aperture and propagate toward the focal region. Yet, for numerical computations, the polar θ and azimuthal angles φ are discretized into $N_{\theta }$ and $N_{\varphi }$ steps, equally spaced by Δθ and Δφ, respectively

$$\left\{ \begin{array}{l} \theta_{1}=0\\ \theta_{m}=\theta_{m-1}+\Delta \theta ,(m\in 1,N_{\theta })\\ \Delta \theta =\alpha /N_{\theta } \end{array},\;\{\begin{array}{l} \varphi_{1}=0\\ \varphi_{n}=\varphi_{n-1}+\Delta \varphi ,(n\in 1,N_{\varphi })\\ \Delta \varphi =2\pi /N_{\varphi } \end{array}.\right.$$ (6)

Consequently, the diffraction integral may be discretized by summing up a finite number of plane waves. This discretized form is more convenient for numerical computation and can be written as

$$E^{\left(3\right)}(x,y,z)=-\frac{ikf}{2\pi }\overset{N_{\theta }}{\underset{m=1}{\sum }}\overset{N_{\varphi }}{\underset{n=1}{\sum }}\Lambda^{\left(3\right)}(\theta ,\varphi )A^{\left(0\right)}(\theta ,\varphi )e^{ik_{0}n_{1}(x\;\sin\;\theta_{1}\;\cos\;\varphi +y\;\sin\;\theta_{1}\;\sin\;\varphi )}\;e^{ik_{0}n_{3}z\;\cos\;\theta_{3}}e^{ik_{0}\Phi^{\left(3\right)}}\;\sin\;\theta \Delta \theta \Delta \varphi .$$ (7)

The focal intensity is then calculated as the squared modulus of the electric field

$$I^{\left(3\right)}=|E^{\left(3\right)}{|}^{2}=|E_{x}{|}^{2}+|E_{y}{|}^{2}+|E_{z}{|}^{2}.$$ (8)

### 3.2 Incident Beam

The incident beam on the input pupil of the objective lens can be written

$$A^{\left(0\right)}=A^{\left(0\right)}(\theta ,\varphi )P^{\left(0\right)}e^{i\Phi (\theta ,\varphi )}e^{iM(\theta ,\varphi )},$$ (9)

where $A^{\left(0\right)}(\theta ,\varphi )$ is the complex amplitude, $P_{0}$ is the polarization of the incident beam, Φ(θ,φ) is the aberration function, and M(θ,φ) is the phase profile of the input beam, corresponding to the phase mask used to shape the beam.

#### 3.2.1 Amplitude

The amplitude of a Gaussian beam incident on the input plane of the objective lens can be written in cylindrical coordinates as

$$A^{\left(0\right)}(\theta ,\varphi )=A_{0}e^{-\frac{\rho^{2}}{w^{2}}},$$ (10)

where $A_{0}$ is a constant and w is the beam waist. Although passing through an aplanatic objective, the incident plane wave transforms into a spherical wave converging to the focal point. Therefore, the amplitude distribution after the objective can be expressed as

$$A^{\left(1\right)}(\theta ,\varphi )=A_{0}e^{-\frac{\rho^{2}}{w^{2}}}\sqrt{\cos\;\theta },$$ (11)

where the cylindrical coordinate on the exit pupil of the objective lens is given by the sine condition^9^ ρ=f sin θ and $\sqrt{\cos\;\theta }$ the apodization term ensuring energy conservation, whereas the beam passes through the objective.

#### 3.2.2 Polarization

Assuming a transverse polarization state of the input field, the incident field can be written

$$P_{0}=(\begin{array}{c} P_{x}^{\left(0\right)}(\theta ,\varphi )\\ P_{y}^{\left(0\right)}(\theta ,\varphi )\\ 0 \end{array}).$$ (12)

Because of the tight focusing, the objective transforms this input transverse polarization into a partially axial polarization in the focal region polarization, which can be described by the 3×3 lens operator matrix $L_{\theta }$.

$$L_{\theta }=(\begin{array}{ccc} \cos\;\theta & 0 & \sin\;\theta \\ 0 & 1 & 0\\ -\sin\;\theta & 0 & \cos\;\theta \end{array}).$$ (13)

Accounting for the interface of the coverslip, the electric field polarization in the sample (3rd medium) can be written as^11^

$$P^{\left(3\right)}(\theta ,\varphi )=\Lambda^{\left(3\right)}(\theta ,\varphi )P^{\left(0\right)}=R_{\varphi }^{-1}[P^{\left(3\right)}{]}^{-1}I^{\left(2\right)}P^{\left(1\right)}L_{\theta_{1}}R_{\varphi }P^{\left(0\right)},$$ (14)

where $R_{\varphi }$ represents the coordinate transformation resulting from a rotation around the z-axis, $P^{\left(l\right)}$ corresponds to the coordinate system rotation in medium l, and $I^{\left(2\right)}$ is the matrix that describes the effect of the coverslip, considered a stratified medium of two interfaces.

$$R_{\varphi }=(\begin{array}{ccc} \cos\;\varphi & \sin\;\varphi & 0\\ -\sin\;\varphi & \cos\;\varphi & 0\\ 0 & 0 & 1 \end{array}),\;P^{\left(l\right)}=(\begin{array}{ccc} \cos\;\theta_{l} & 0 & -\sin\;\theta_{l}\\ 0 & 1 & 0\\ \sin\;\theta_{l} & 0 & \cos\;\theta_{l} \end{array})\;\text{and}\;I^{\left(2\right)}=(\begin{array}{ccc} T_{p}^{\left(2\right)} & 0 & 0\\ 0 & T_{s}^{\left(2\right)} & 0\\ 0 & 0 & T_{p}^{\left(2\right)} \end{array}).$$ (15)

where $\theta_{l}$ is the focusing angle in the l’th medium and $T_{s,p}^{\left(2\right)}$ are the transmission coefficients in the coverslip.

In the general case, determining the reflection and transmission coefficients through a stratified medium involves solving Maxwell’s equations. However, in the case of a coverslip with only two interfaces, this simplifies the well-determined Fresnel coefficients for s- and p-polarization

$$T_{p}^{\left(2\right)}=\frac{t_{s,p}^{\left(1\right)}t_{s,p}^{\left(2\right)}e^{ik_{2}t\;\cos\;\theta_{2}}}{1+r_{s,p}^{\left(1\right)}r_{s,p}^{\left(2\right)}e^{ik_{2}t\;\cos\;\theta_{2}}},$$ (16)

with t as the thickness of the coverslip and $t_{s,p}^{\left(j\right)}$ and $r_{s,p}^{\left(j\right)}$ as the amplitude transmission and reflection coefficient at the j’th interface, respectively

$$t_{s}^{\left(j\right)}=\frac{2n_{j}\;\cos\;\theta_{j}}{n_{j}\;\cos\;\theta_{j}+n_{j+1}\;\cos\;\theta_{j+1}}\;t_{p}^{\left(j\right)}=\frac{2n_{j}\;\cos\;\theta_{j}}{n_{j+1}\;\cos\;\theta_{j}+n_{j}\;\cos\;\theta_{j+1}}\;r_{s}^{\left(j\right)}=\frac{n_{j}\;\cos\;\theta_{j}-n_{j+1}\;\cos\;\theta_{j+1}}{n_{j}\;\cos\;\theta_{j}+n_{j+1}\;\cos\;\theta_{j+1}}\;r_{p}^{\left(j\right)}=\frac{n_{j+1}\;\cos\;\theta_{j}-n_{j}\;\cos\;\theta_{j+1}}{n_{j+1}\;\cos\;\theta_{j}+n_{j}\;\cos\;\theta_{j+1}},$$ (17)

with $n_{j}$ and $\theta_{j}$ as the refractive index and the incident angle in the j’th medium, respectively.

For example, in the case of left-handed circular polarization

$$P^{\left(3\right)}(\theta ,\varphi )=(\begin{array}{c} T_{p}^{\left(2\right)}\;\cos\;\theta_{3}\;\cos^{2}\;\varphi +T_{s}^{\left(2\right)}\;\sin^{2}\;\varphi \\ T_{p}^{\left(2\right)}\;\cos\;\theta_{3}\;\cos\;\varphi \;\sin\;\varphi -T_{s}^{\left(2\right)}\;\cos\;\varphi \;\sin\;\varphi \\ -T_{p}^{\left(2\right)}\;\sin\;\theta_{3}\;\cos\;\varphi \end{array})+i(\begin{array}{c} T_{p}^{\left(2\right)}\;\cos\;\theta_{3}\;\sin\;\varphi \;\cos\;\varphi -T_{s}^{\left(2\right)}\;\sin\;\varphi \;\cos\;\varphi \\ T_{p}^{\left(2\right)}\;\cos\;\theta_{3}\;\sin^{2}\;\varphi +T_{s}^{\left(2\right)}\;\cos^{2}\;\varphi \\ -T_{p}^{\left(2\right)}\;\sin\;\theta_{3}\;\cos\;\varphi \end{array}).$$ (18)

#### 3.2.3 Optical aberrations

In Eq. (9), Φ(θ,φ) represents the wavefront distortion relative to the reference sphere, an effect commonly called optical aberration. A convenient approach is to express these distortions in cylindrical coordinates on the objective pupil and to decompose them using Zernike polynomials:

$$\Phi (\rho ,\theta )=\sum_{n,m}C_{n}^{m}Z_{n}^{m}(\rho ,\theta ),$$ (19)

where n∈N is the radial degree, m∈Z is the azimuthal degree, and $C_{n}^{m}$ and $Z_{n}^{m}$ are the Zernike coefficients and polynomials, respectively.^19^^,^^20^

$$Z_{n}^{m}(\rho ,\theta_{c})=R_{n}^{m}(\rho )G^{m}(\theta_{c}),$$ (20)

with

$$R_{n}^{m}(\rho )=\overset{(n-m)/2}{\underset{l=0}{\sum }}\frac{(-1{)}^{l}(n-l)!}{l![\frac{1}{2}(n+m)-l]![\frac{1}{2}(n-m)-l]!}r^{n-2l},\;G^{m}(\theta_{c})=\{\begin{array}{cc} \cos(m\theta_{c}) & \text{if}\;m\geq 0\\ \sin(m\theta_{c}) & \text{if}\;m<0 \end{array}.$$ (21)

These modes are normalized to $R_{n}^{m}(1)=1$ which was introduced by Noll in 1975.^21^

Zernike polynomials are widely employed to model optical aberrations due to their orthogonality (on the unit sphere). In the paraxial approximation, each polynomial function corresponds to a distinct type of optical aberration, such as astigmatism, coma, or spherical.^19^^,^^20^

#### 3.2.4 Phase mask

Spatial shaping of the focal field distribution is often achieved using a phase mask M(θ,φ), imprinted on the incident laser beam prior to reaching the objective lens.

Various phase masks are available to achieve different spatial shapes. For convenience, the phase mask is expressed in spherical coordinates.

**Table:** 

Gaussian	Donut	Bottle
None	Vortex mask	Ring mask
M(θ,φ)=0	M(θ,φ)=qφ	$M(\theta ,\varphi )=\{\begin{array}{ll} q\pi & \text{for}\;\theta \leq \theta_{M}\\ 0 & \text{for}\;\theta_{M}\leq \theta \leq \alpha \end{array}$

where q∈R is an integer representing the topological charge of the vortex or ring, respectively. To produce a bottle beam, q has to be an odd number. $\theta_{R}$ is the angle between the optical axis and the edge marginal ray of the π-phase ring of the phase mask.

$$\theta_{R}=\arcsin(\frac{r_{R}}{r_{\mathrm{pup}}}\;\sin\;\alpha ),$$ (22)

with $r_{R}$ the radius of the π-phase ring and $r_{\mathrm{pup}}$ the radius of the front pupil of the objective.

Finally, in the case of donut and bottle beam shaping, which are used in 3D-STED microscopy, the two PSFs are treated separately and added in the far field, assuming orthogonal polarization using a weighted sum

$$I_{\mathrm{tot}}=pI_{\text{donut}}+(1-p)I_{\text{bottle}},$$ (23)

with p∈[0,1] so that p=0 and p=1 correspond to a donut- and bottle-shaped beam, respectively.

### 3.3 Effective Fluorescence PSF

In the case of STED microscopy, the effective fluorescent spot results from the combined action of the excitation PSF $I_{e}$ (e.g., confocal, two-photon) and the de-excitation PSF $I_{d}$ (e.g., donut, bottle). In FASER, both are computed independently and the effective fluorescent PSF $I_{\text{eff}}$ is determined in every pixel as^22^

$$I_{\mathrm{eff}}=I_{e}e^{-\ln\;2.\frac{I_{d}}{I_{\mathrm{sat}}}},$$ (24)

where $I_{\mathrm{sat}}$ represents the saturation factor accounting for the interaction between the STED laser (de-excitation) and the fluorophore.

### 3.4 Focusing Geometry

#### 3.4.1 Impact of the cranial window

In many cases, optical access to the sample occurs via a coverslip, which serves as a layer of mutual protection for the microscope and the sample. This is a common methodology in neuro-imaging, where a “cranial window” literally means a miniature fenestration in the skull of the animal that is sealed by a thin glass to avoid physical leakage in and out of the interior.^23^ It makes it possible to maintain the brain in physiological conditions while providing high-quality optical access for extended periods over the lifetime of the animal. To simulate this experimental configuration, we introduced an additional amplitude mask

$$\mathrm{CW}(\theta ,\varphi )=\{\begin{array}{lll} 1 & \text{for}\;\theta \leq \theta_{c} & \text{transmitted rays}\;\\ 0 & \text{for}\;\theta_{c}\leq \theta \leq \alpha & \text{blocked region} \end{array}.$$ (25)

where $\theta_{c}$ is the angle between the optical axis and the marginal ray of the cranial window on the output pupil of the objective. For further details, refer to Ref. 7.

In the diffraction integral, this simply leads to a change of the integration limit to account for the reduced effective pupil size.

#### 3.4.2 Case of a tilted coverslip

In real-world conditions, orthogonality of the coverslip with respect to the optical axis can be challenging to achieve and a few degrees of deviation can easily occur, particularly in the case of *in vivo* imaging. A tilted interface breaks the rotational symmetry of the previous configuration and is therefore expected to affect the phase of the laser beam. Apart from spherical aberrations induced by the stratified medium from the objective to the sample, a tilted coverslip introduces significant aberrations, especially coma. However, only a few studies deal with this case, most of them using undisclosed algorithms. In FASER, we implemented an approach proposed by Berning^24^ and further detailed in Ref. 25 to extend the theoretical framework presented in Sec. 2.2 to this new geometry.

Schematically, tilting the coverslip by an angle γ is equivalent to rotating the objective lens, and the incident beam, by an angle −γ. However, analytically, it is simpler to derive the focal field using the rotated objective lens method. Indeed, tilting the coverslip requires modifying Eq. (4), and notably, the integration range and the boundary conditions. By contrast, the second method can simply be realized through a transformation of the pupil function to rotate the spherical wavefront $A^{\left(1\right)}$ around the origin 0. The integration limit on θ is expanded to α+|γ|, to ensure the entire wavefront is accounted for without clipping, while the amplitude of the beam falling out of the pupil is set to 0. The new pupil function $A_{\gamma }^{\left(0\right)}(\theta ,\varphi )$ is essentially the pupil function of the untitled case but evaluated at the transformed position $A^{\left(0\right)}(\theta^{\prime },\varphi^{\prime })$. The rotated coordinate can be expressed as follows:

$$\theta^{\prime }=a\sin(\frac{\sqrt{x^{\prime 2}+y^{\prime 2}}}{f}),\;\varphi^{\prime }=\mathrm{atan}(\frac{x^{\prime }}{y^{\prime }}),$$ (26)

with

$$(\begin{array}{c} x^{\prime }\\ y^{\prime } \end{array})=f(\begin{array}{c} \cos\;\gamma \;\cos\;\varphi \;\sin\;\varphi -\sin\;\gamma \;\cos\;\theta \\ \sin\;\varphi \;\sin\;\theta \end{array}).$$ (27)

All parameters related to the objective lens, and hence to its coordinate system, have to be transformed in this way, notably the apodization factor and the phase correction term [Eq. (5)].

$$E_{\text{tilt}}^{\left(3\right)}(x,y,z)=-\frac{ikf}{2\pi }\int_{0}^{\alpha +|\gamma |}\int_{0}^{2\pi }\sqrt{\cos\;\theta^{\prime }}\Lambda^{\left(3\right)}(\theta ,\varphi )A^{\left(0\right)}(\theta^{\prime },\varphi^{\prime })CW(\theta ,\varphi )e^{i\Phi (\theta^{\prime },\varphi^{\prime })}e^{iM(\theta^{\prime },\varphi^{\prime })}\;e^{ik_{0}n_{1}(x\;\sin\;\theta_{1}\;\cos\;\varphi +y\;\sin\;\theta_{1}\;\sin\;\varphi )}e^{ik_{0}n_{3}z\cos\theta_{3}}e^{ik_{0}(n_{3}d\cos\theta_{3^{\prime }}-n_{1}(t+d)\cos\theta_{1^{\prime }})}\sin\;\theta d\theta \;d\varphi .$$ (28)

## 4 Installation

**Fig. 2:**
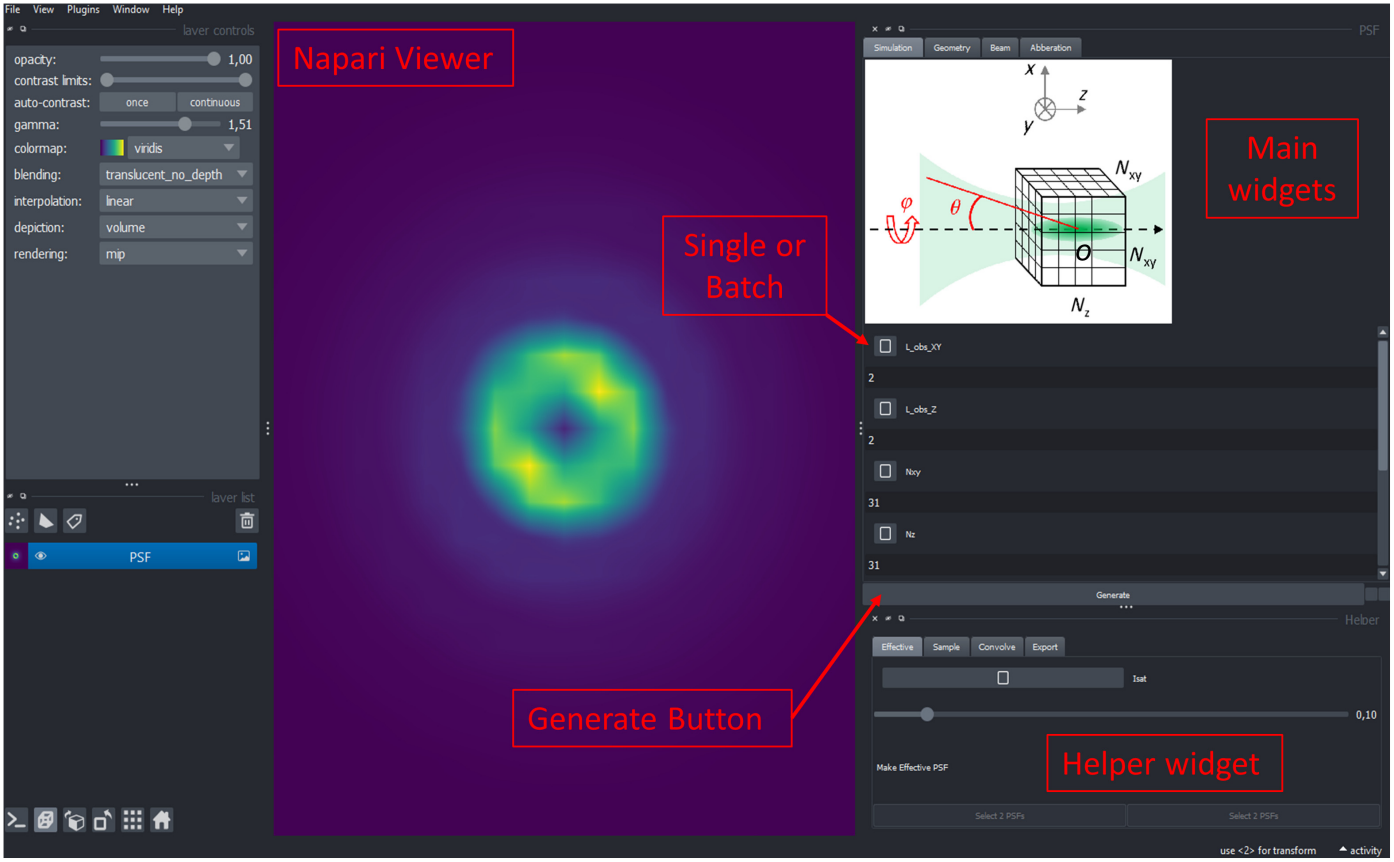
FASER GUI. Based on the Napari viewer, FASER features a main widget window with multiple tabs to set the simulation parameters and the property of the input beam and define the focusing geometry. A helper widget window provides additional tabs to compute the effective PSF in STED microscopy, simulate an artificial object, convolve it with the PSF, and export the simulated stacks of images.

FASER provides high-level functions for configuring and running simulations of focused light fields and was written as a Napari plugin. Its graphical user interface (GUI) is depicted in Fig. 2. We offer three primary ways to use FASER, each intended to support different use cases.

• First, FASER is available as a plugin directly through the Napari Plugin Hub. This makes it straightforward to install into an existing Napari environment without requiring programming knowledge. The plugin can be found in Ref. 26.
• Second, FASER can be installed as a standalone tool in any Python 3.10 environment by running pip install faser napari[pyqt5]. It can be launched using qtfaser. This approach includes a command-line interface, enabling its integration into automated computational workflows-for instance, to run multiple simulations while exploring a larger parameter space.
• Third, FASER can be installed directly from its source code hosted at https://github.com/jhnnsrs/faser. To do so, first, create a new environment with Python 3.10.11 and install Napari pip install napari. Then, clone the FASER repository using git clone https://github.com/jhnnsrs/faser.git, navigate to the cloned directory with cd faser, and install FASER using pip install to ensure all dependencies are installed. Finally, start Napari by running python nap.py.

## 5 FASER Design and Implementation—Example Case of STED Microscopy

The objective of FASER is to provide an open-access, educational tool for simulating the excitation PSF of a microscope. The GUI simplifies setting calculation parameters that define the simulation, and key parameters including the input beam, the geometry of focalization, and the numerical computation parameters.

As an example, in the following sections, we focus on FASER’s capability to simulate toroidal PSFs used in STED microscopy. However, as detailed in Secs. 2 and 3, FASER is based on vectorial diffraction theory, which offers a general framework for computing the PSF of any optical system. Therefore, the examples presented here can be extended to other microscopy techniques. It is important to note that currently, FASER only considers excitation PSFs. Other parameters, such as pinhole sizes that come into play in confocal microscopy, would need to be included to compute the emission PSF.

### 5.1 Main Widgets

The main widget contains four tabs designed for configuring simulated parameters, input beam properties—including potential aberrations stemming from flawed optics and setup alignment before the objective lens—and setting the focalization geometry under consideration. Unless otherwise specified, default values set in FASER were applied for the calculation.

#### 5.1.1 Simulation parameters

The first tab is used to set simulation parameters such as the number of pixels in X, Y, and Z directions, along with the observation scale. In addition, the number of integration steps in φ and θ angles are defined here. A check box allows normalization of the maximum pixel value of the PSF intensity to 1. Beyond default values, all these parameters can be independently set prior to launching the simulations.

**Fig. 3:**
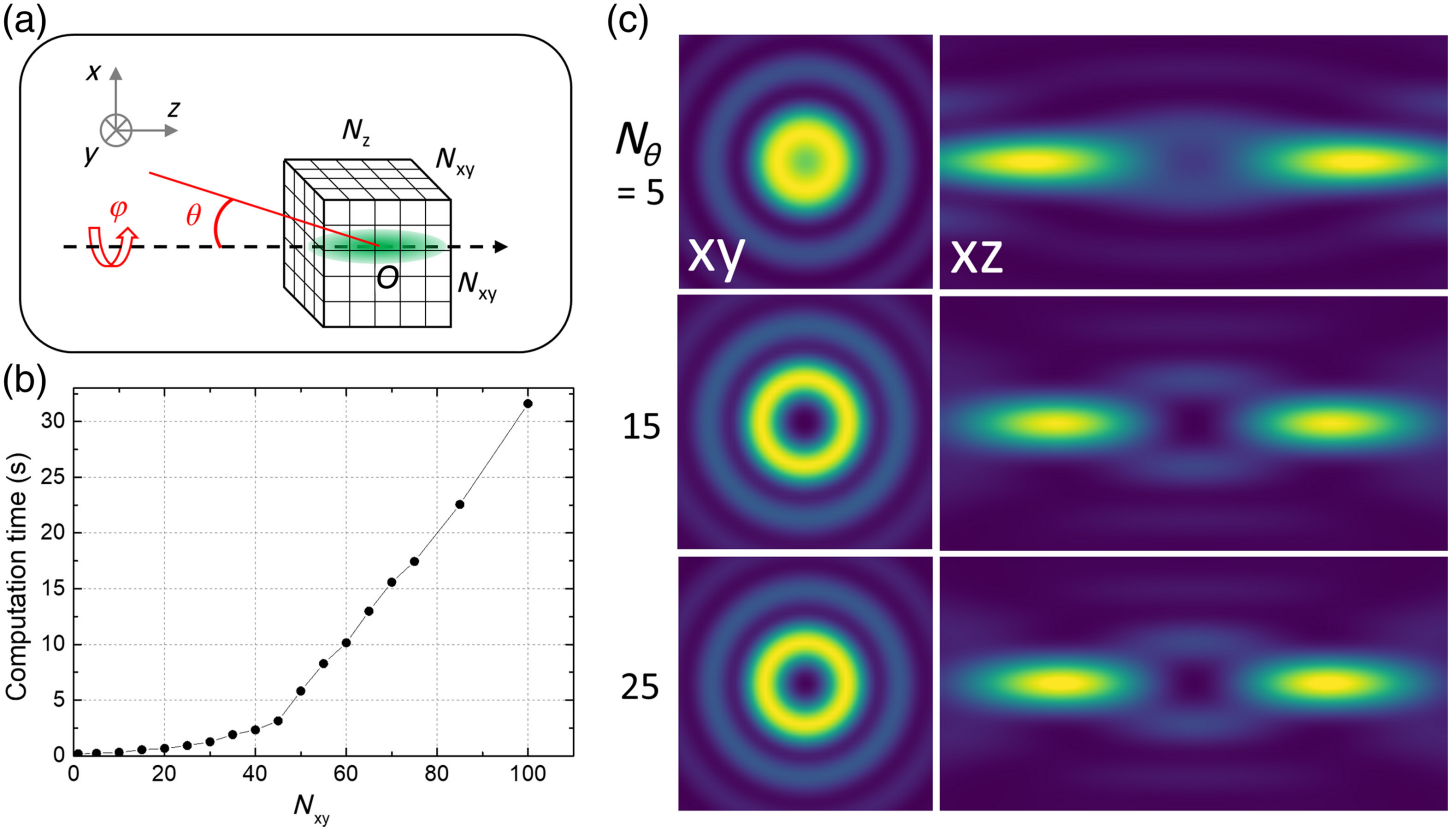
Simulation parameters. (a) Schematic of the simulation configuration. (b) Computation time as a function of the number of XY pixels in the computed volume. (c) Lateral ($2\times 2\;\mu m^{2}$) and axial ($2\times 4\;\mu m^{2}$) views of a *bottle-shaped* PSF for different θ integration steps.

Figure 3 illustrates the effects of these parameters on computation time and PSF accuracy. As expected, computation time increases quadratically with the number of pixels in the X and Y directions (defined as a common parameter: $N_{XY}$). Conversely, changing the number of integration steps $N_{\theta }$ from 5 to 15 strongly impacts the simulated PSF, particularly the quality of the zero in the center. Increasing the number of steps has a more limited impact on the results. As a compromise between speed and accuracy, the default values of pixel number and integration steps are set to 31. However, it is worth noting that the optimal number of integration steps depends on the complexity of the phase mask and the aberration profile of the incident beam. Through various configurations, we found a good compromise between computation time and number of pixels and steps. Yet, simpler PSFs (such as a Gaussian beam) can be appropriately computed with fewer steps, whereas highly structured PSFs might fare better with a higher number of pixels and steps.

To facilitate efficient computation in the Python environment, FASER employs strategies of Just-In-Time Compilation (utilizing Numba) and employs lazy compute patterns through Dask. The computing times provided in the next section have been obtained on a standard desktop running on Windows 11 Pro 64 bits, equipped with an Intel core i5-1335U processor (1.30 GHz) and 16 GB of RAM.

#### 5.1.2 Focusing geometry

**Fig. 4:**
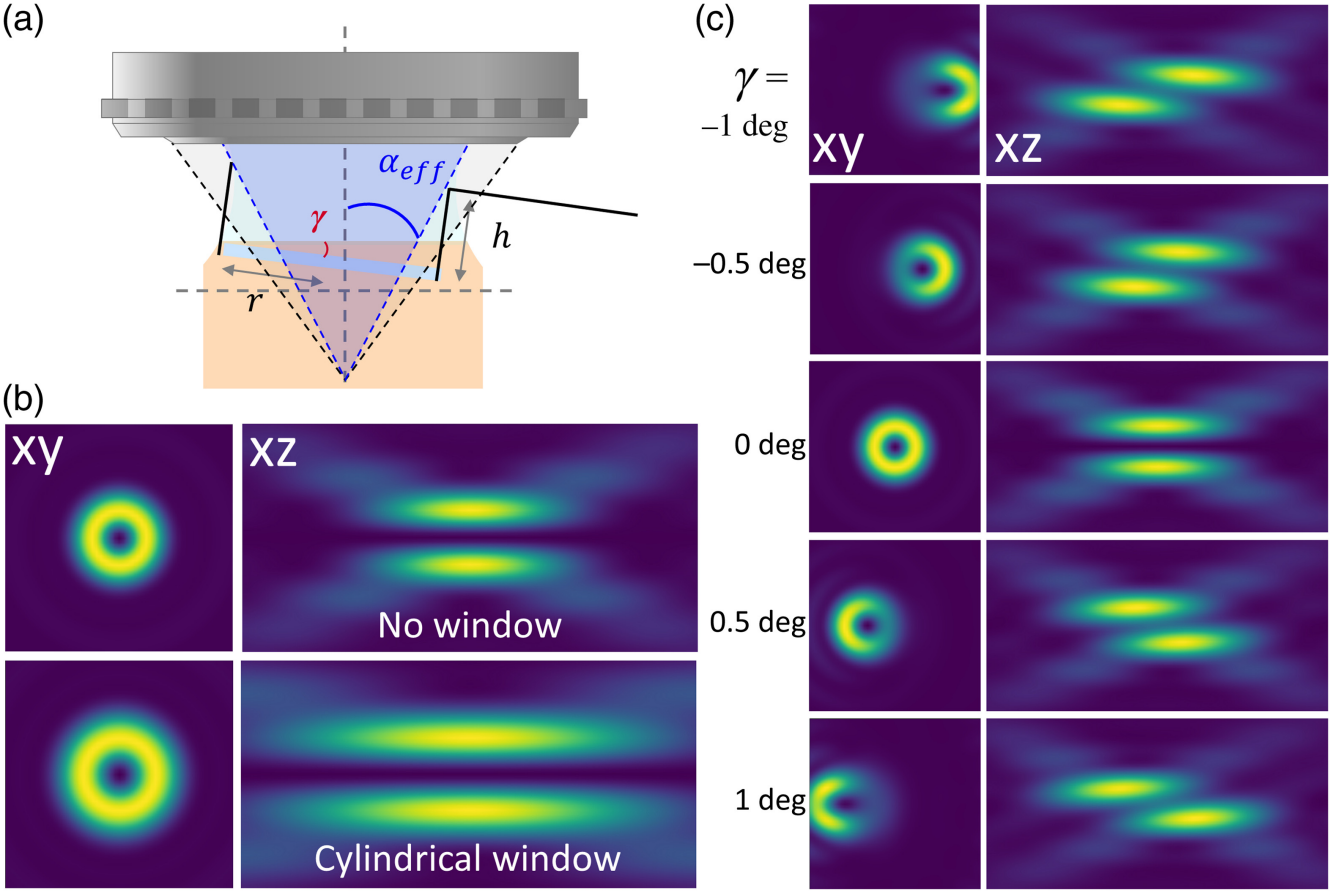
Focusing geometry. (a) Schematic of the optical geometry after the microscope objective. (b) PSF simulation with and without a cylindrical cranial window.^27^ (c) Impact of a tilted coverslip on the *donut-beam* profile. Image size $2\times 2\;\mu m^{2}$ for XY views and $2\times 4\;\mu m^{2}$ for XZ views.

The second tab serves to define the focalization geometry under consideration, as depicted in Fig. 4. This includes specifying the objective parameters (NA, working distance, refractive index of the immersion media and correction collar), details about the coverslip (refractive index, thickness, tilt), and the characteristics of the sample (refractive index, depth). In addition, to account for a potential cranial window used for *in vivo* imaging, an aperture is added right after the objective lens.

Figure 4(b) illustrates the impact of a cylindrical cranial window. As expected, the window reduces the effective numerical aperture, leading to an extended focus. The impact of the cranial window geometry in STED microscopy has been previously described.^27^ In addition, a tilt angle of the coverslip introduces severe distortions of the PSF as reported.^25^ This parameter is particularly critical *in vivo*, where tilt angles ∼1  deg can inadvertently be introduced.

#### 5.1.3 Input beam parameters

##### Intensity distribution, polarization, and phase mask

**Fig. 5:**
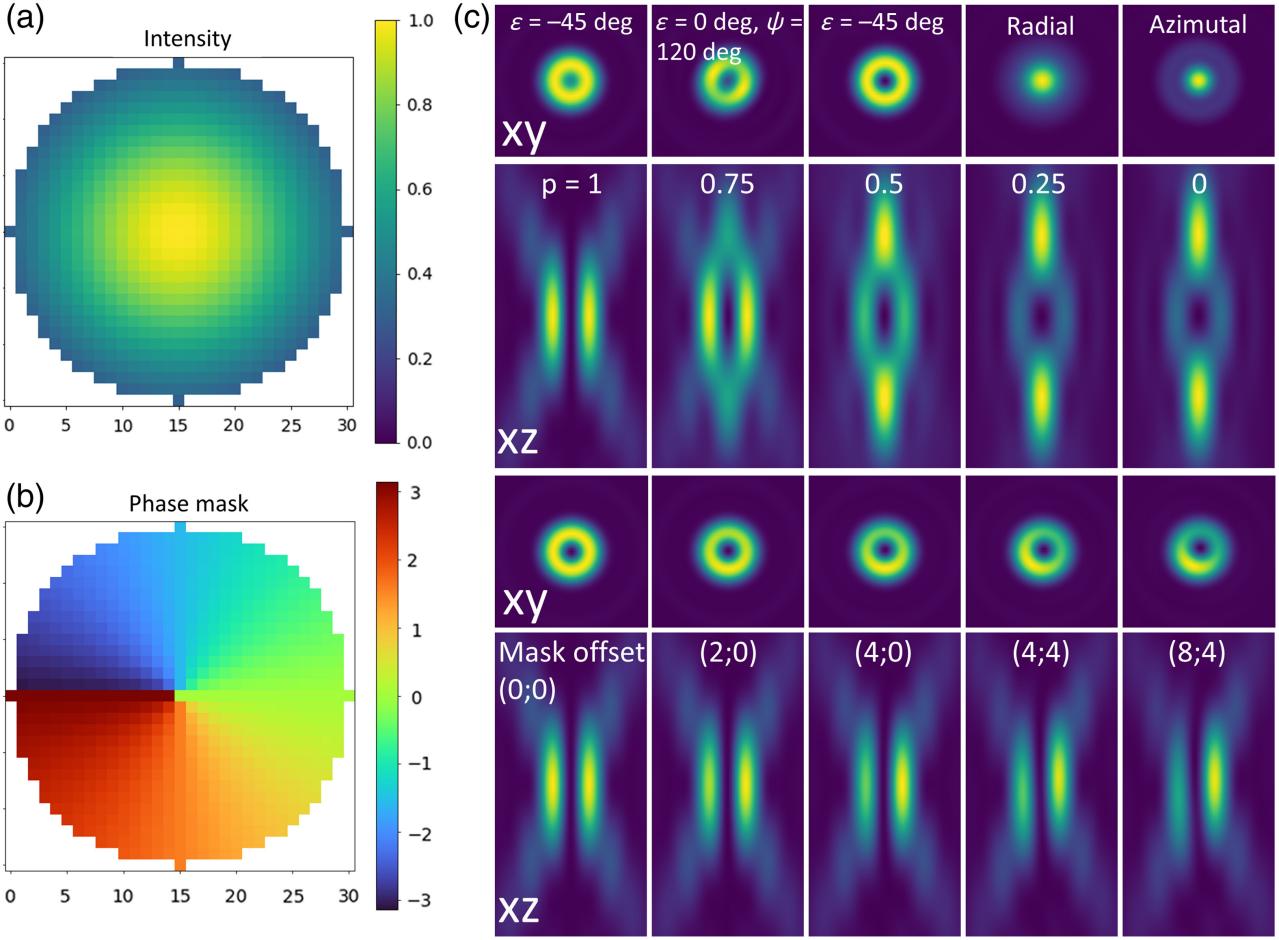
Input beam. (a) Pop-up window displaying the intensity profile on the back pupil of the objective. (b) Pop-up window showing the phase mask imprinted on the input beam. (c) Effect of the polarization on donut beam PSF (in-plane views - $2\times 2\;\mu m^{2}$). (d) Donut and bottle profiles at different p ratios (axial views - $2\times 4\;\mu m^{2}$. (e) Impact of misaligned vortex mask on the donut-shaped PSF.

The third tab in the main widget is used to define the properties of the input beam. The intensity profile incident on the input pupil of the objective is defined as a Gaussian profile with a specified wavelength and waist. In addition, a misalignment of the input beam can be modeled through an offset of the intensity profile. A “show intensity” push-button allows visualization of the input profile on the back pupil of the objective lens [see Fig. 5(a)].

FASER also allows the user to set the polarization of the beam. A toggle box allows selection between elliptical (default), radial, or azimuthal polarization. For elliptical polarization, ϵ control the ellipticity (−45  deg right-handed circular, 0 deg linear, and 45 deg left-handed circular) and Ψ the polarization direction.

Furthermore, a phase mask can be applied to the input beam. A toggle box allows the selection of desired profiles (Gaussian, donut, bottle, or donut and bottle). Alternatively, a custom-defined phase mask can be loaded. Donut beams are formed using a vortex phase mask defined by an integer vortex charge. Bottle beams are generated with a ring phase mask defined by the ring radius and charge. In both cases, an offset can be introduced to model a misalignment of the phase mask with respect to the optical axis of the objective. Last, a combination of donut and bottle beams, commonly used in 3D-STED microscopy, can be modeled with a parameter p indicating the intensity split between the two-phase modulations. Once again, a “show phase mask” push-button enables to visualize the imprinted mask on the back pupil of the objective lens [see Fig. 5(b)].

Figure 5 illustrates the impact of different parameters (polarization, phase mask offset) on the donut beam PSF. It illustrates that mask misalignment can easily be mistaken for optical aberrations, as previously investigated and quantified.^28^^,^^29^ In addition, a detailed study of the effect of STED beam polarization can be found in Hao et al.^30^

##### Optical aberrations

**Fig. 6:**
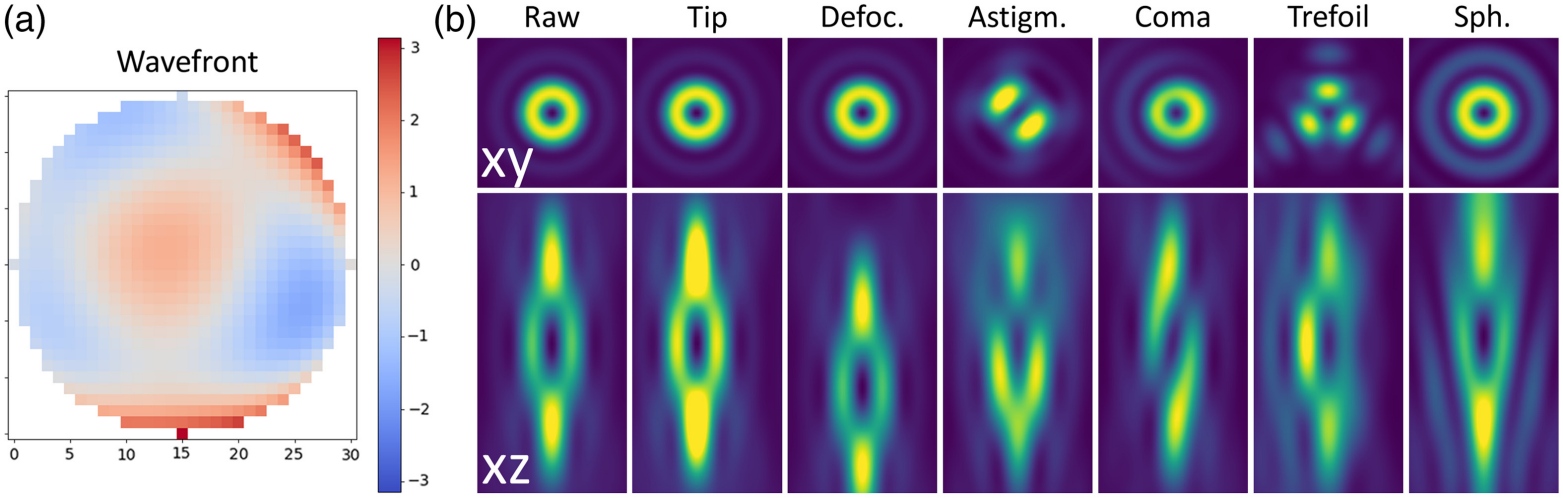
Optical aberrations. (a) Pop-up window showing an example of an aberrated wavefront (a mixture of aberrations) on the back pupil of the objective. (b) Impact of the main aberrations on donut and bottle PSFs (with p=0.5). Lateral views $2\times 2\;\mu m^{2}$ and axial views $2\times 4\;\mu m^{2}$.

Beyond the phase mask, to model a realistic optical system, optical aberrations can be added to the incident beam. We focused on the first-order aberrations (tip, tilt, astigmatism, Coma, trefoil, and spherical) as well as second-order spherical aberration, as they represent the dominant aberrations in optical microscopy. The fourth and final tab of the main widget allows independent setting of the coefficients for these 11 modes, as well as an offset of the aberrated wavefront. A push-button “show aberrations” enables visualization of the input profile on the back pupil of the objective lens [see Fig. 6(a) for an example].

Figure 6 illustrates the effect of the most classical aberrations on the mixture of donut and bottle beam profiles.

### 5.2 Helper Widgets

**Fig. 7:**
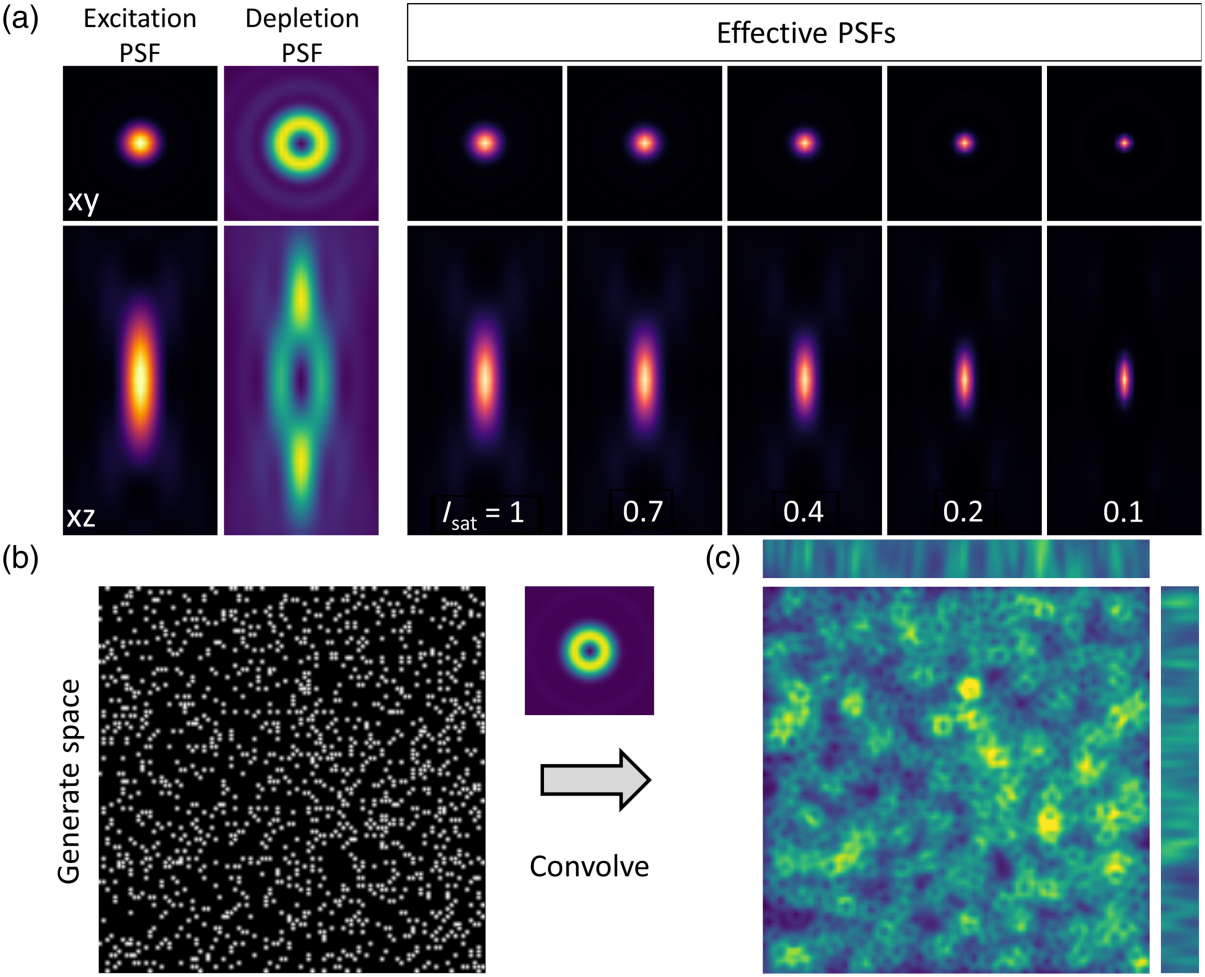
Helper widget. (a) Example of an effective PSF calculated from the excitation and depletion PSFs (left panels) for various saturation factors. (b) Example of a generated space ($100\times 100\times 10\;\mu m^{3}$) with 1000 spots. (c) Convolution with the donut and bottle PSF.

As explained above, the main widget serves to define the simulation parameters for the PSFs. FASER also incorporates several helper widgets, providing additional functionality (Fig. 7).

The first tab enables the computation of the effective PSF for the case of STED microscopy. The user has to select two previously simulated PSFs: one for excitation and one for depletion. The effective PSF is computed using the defined saturation factor according to Eq. (24).

A sample tab enables the simulation of an artificial sample of defined size in all three dimensions, with a specified number of points randomly distributed. The next tab serves to convolve images. It is originally intended to convolve a simulated PSF with a sample space, illustrating how a modified PSF impacts the image. Additional structures (line grid and concentric circles) were added to illustrate the impact of PSF distortion on the particular features in the images.

An export tab makes it possible to save a z-stack of the simulated PSF as a.tiff file. This includes saving the simulation parameters as metadata accompanying the data.

Last, FASER includes a metric tab with a “calculate maximum” push-button that creates a pop-up window with the maximum intensity in the selected PSF. This is useful for batch mode computation, making it possible to graphically visualize the impact of one (lineplot) or two (heat map) variable parameter(s) on the intensity of the PSF.

## 6 Conclusion

In summary, we present FASER, a Python-based software package for simulating microscopy PSFs using fully vectorial calculations. FASER is equipped with a comprehensive set of features that allows users to configure key optical parameters of the input beam (such as polarization and aberrations), as well as geometrical parameters related to the sample. In addition, it supports the simulation of wavefront shaping through the introduction of a phase mask. The software is intended as a pedagogical tool, enabling users to explore and understand the impact of potential misalignments on microscope performance, with the goal to help identify the most important parameters that influence imaging quality.

## Data Availability

The archived version of the code described in this paper can be freely accessed through https://github.com/jhnnsrs/faser.
